# Left ventricular non-compaction: clinical features and cardiovascular magnetic resonance imaging

**DOI:** 10.1186/1471-2261-9-37

**Published:** 2009-08-09

**Authors:** Zaheer R Yousef, Paul WX Foley, Kayvan Khadjooi, Shajil Chalil, Harald Sandman, Noor UH Mohammed, Francisco Leyva

**Affiliations:** 1 Department of Cardiology, Good Hope Hospital, Sutton Coldfield, West Midlands, UK

## Abstract

**Background:**

It is apparent that despite lack of family history, patients with the morphological characteristics of left ventricular non-compaction develop arrhythmias, thrombo-embolism and left ventricular dysfunction.

**Methods:**

Forty two patients, aged 48.7 ± 2.3 yrs (mean ± SEM) underwent cardiovascular magnetic resonance (CMR) for the quantification of left ventricular volumes and extent of non-compacted (NC) myocardium. The latter was quantified using planimetry on the two-chamber long axis LV view (NC area). The patients included those referred specifically for CMR to investigate suspected cardiomyopathy, and as such is represents a selected group of patients.

**Results:**

At presentation, 50% had dyspnoea, 19% chest pain, 14% palpitations and 5% stroke. Pulmonary embolism had occurred in 7% and brachial artery embolism in 2%. The ECG was abnormal in 81% and atrial fibrillation occurred in 29%. Transthoracic echocardiograms showed features of NC in only 10%. On CMR, patients who presented with dyspnoea had greater left ventricular volumes (both p < 0.0001) and a lower left ventricular ejection fraction (LVEF) (p < 0.0001) than age-matched, healthy controls. In patients without dyspnoea (n = 21), NC area correlated positively with end-diastolic volume (r = 0.52, p = 0.0184) and end-systolic volume (r = 0.56, p = 0.0095), and negatively with EF (r = -0.72, p = 0.0001).

**Conclusion:**

Left ventricular non-compaction is associated with dysrrhythmias, thromboembolic events, chest pain and LV dysfunction. The inverse correlation between NC area and EF suggests that NC contributes to left ventricular dysfunction.

## Background

Before the fifth week of intrauterine life, the myocardium forms a loose network of fibers and sinusoids which are in continuity with the ventricular cavity. Subsequently, the meshwork of fibers becomes 'compacted' and the sinusoids disappear. Pathological arrest of this compaction process leads to the persistence of ventricular hypertrabeculation, so called spongy myocardium or left ventricular (LV) non-compaction (NC). [1]

In both children and adults, NC is related to a cardiomyopathy which is hitherto unclassified. [2] The childhood form of NC was first described in association with other congenital abnormalities, such as cyanotic congenital heart disease, coronary artery anomalies and both right and LV outflow tract obstruction. [3-5] Furthermore, it may be associated with neuromuscular abnormalities. In 1990, Chin et al described a group of 8 patients with NC which was not associated with other congenital cardiac abnormalities. [6] Whilst some reports indicate that NC is a rare condition with a poor prognosis, [3,7,8] others suggest that it is more common and that its prognosis is better than expected[9]Application of the current diagnostic criteria may identify a significant pool of patients who are asymptomatic. [10] Although NC has generally been regarded as a familial cardiomyopathy, [11] a family history of cardiomyopathy is not always present in adults [7,9] or children [8] with the morphological characteristics of NC. Given that NC can be present in asymptomatic individuals, Murphy et al have hypothesised that the condition may have a long pre-clinical phase. [9] Interestingly, there are anecdotal reports of cases of patients with phenotypic non-compaction which resolves with standard heart failure treatment. [12]

Most studies have employed echocardiography for the identification of NC. [3,7,9] Recently, however, an increasing number of case reports [13-16] and series [11] of patients with NC have demonstrated the superiority of cardiovascular magnetic resonance (CMR) over echocardiography in the assessment of NC. However, tere is only one report where the CMR findings were confirmed with direct visualisation of the myocardium. [15] We report the clinical features and CMR findings of 42 patients who were consecutively identified as having morphological features of NC.

## Methods

### Patients

Patients were studied following referral to a regional CMR service over a 4 year period. The service has a special interest in heart failure and cardiomyopathy. Information regarding presenting symptoms and family history was obtained retrospectively from case notes and patient interviews. All patients underwent a 12-lead ECG and a CMR scan, which is regarded as the gold standard investigation for heart failure. [17] Echocardiographic details as well as the reason for referral for the initial transthoracic echocardiogram were obtained from referring centres. Left ventricular hypertrophy (LVH) was defined as a left ventricular wall thickness > 13 mm. Left ventricular systolic dysfunction (LVSD) was defined as an LVEF ≤ 40%.

The control group comprised 22 subjects who were referred for CMR for the exclusion of a variety of abnormalities, but who were found to have a normal study. Controls were selected to match the age and sex of the subjects. Transthoracic echocardiograms were obtained with Vivid 5 and Vivid 7 (GE, Slough, UK) machines and analysed with EchoPac (GE, Slough, UK). Left ventricular volumes were measured from the apical 4 chamber view, and LV ejection fraction using the modified Simpson's technique. [18]

### Cardiovascular magnetic resonance

Images were acquired on a 1.5 Tesla (General Electric Signa) scanner using a phased array cardiac coil during repeated 8-second breathholds. A short axis stack of LV images was acquired using a steady state in free precession (SSFP) sequence (repetition time 3.0 to 3.8 ms; excitation time 1.0 ms; image matrix 224 × 224; field of view 36–42 cm; flip angle 45°) in sequential 8 mm slices (2 mm interslice gap) from the atrioventricular ring to apex. Left ventricular volumes, ejection fraction and mass (myocardial density = 1.05 g/cm^3^) were quantified using manual planimetry of all short-axis SSFP cine images with MASS analysis software (Medis, the Netherlands) The non-compacted myocardium was excluded from the analysis, and was easily distinguished on CMR. The papillary muscles were excluded from the analysis of left ventricular volumes, as were moderator and aberrant bands.

**Figure 1:**
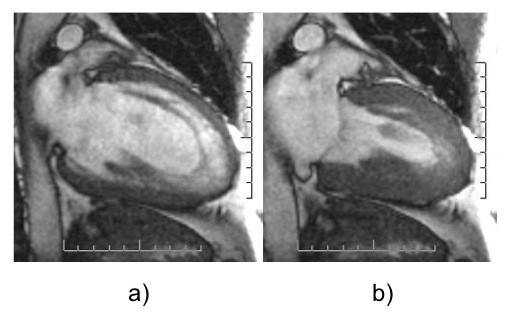
**a) End-diastolic cardiac magnetic resonance two-chamber view of a patient with left ventricular non-compaction; b) two-chamber view showing obliteration of myocardial recesses in systole**.

**Figure 2:**
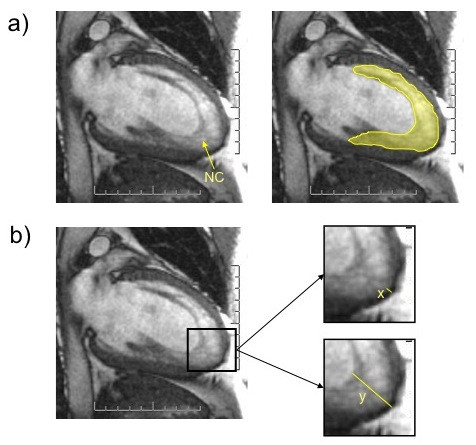
**a) The extent of non-compacted myocardium is quantified using planimetry of the area of non-compacted myocardium (NC) on the two-chamber view; d) the x:y ratio at the apex, as described by Chin et al **[6]**, refers to the ratio of distance between epicardial surface and trough of the recesses (x) to the distance between the epicardial surface and the peak of the trabeculations (y)**.

To quantify the extent of NC, Jenni et al [2] adopted the end-systolic ratio of non-compacted (NC) to compacted (C) myocardial thickness in short axis transthoracic echocardiographic views. In our experience with CMR, however, imaging in the vertical long axis (2-chamber) view offers a superior spatial resolution, perhaps because trabeculae are aligned transversely to the imaging plane (Figure 1a). As Petersen et al [11], we too have found that with CMR, the differentiation between NC and C is maximal at end-diastole (Figure 1a). Systole has the effect of obliterating the sinusoids (Figure 1b). We have therefore used the vertical long axis (2-chamber) end-diastolic view for the quantification of NC. Apical segments were also included in the assessment of NC, as these were the most frequently involved. [11] Selected cine loops were exported into a dedicated image analysis software package (Osirix, freeware from ). After calibration, the following indices of NC were derived: a) NC area, as the area in cm^2 ^of non-compacted myocardium on the two-chamber long axis LV view. This is calculated using planimetry of the area delimited by the peaks of the trabeculation and the troughs of the recesses. (Figure 2a); and b) The x:y ratio at the apex, as described by Chin et al [6], which refers to the ratio of distance between epicardial surface and trough of the recesses (x) to the distance between the epicardial surface and the peak of the trabeculations (y) (Figure 2b). All patients included in this study had an x:y ratio ≥ 0.5.

### Statistical analysis

Continuous variables are expressed as mean ± standard error of the mean (SEM). Comparisons between normally distributed continuous variables were made using the unpaired Student's t test. Pearson correlation analyses were used for univariate correlations. Standard statistical analyses were performed using Statview (Cary, NC, USA) including ANOVA for comparison between groups. A two-tailed p value of < 0.05 was considered statistically significant. The study was approved by Birmingham, North East and Solihull Research Ethics Committee.

## Results

A total of 42 cases of NC were identified over a 4 year period, during which our CMR Unit undertook 763 studies directed at the assessment of LV function and/or myocardial viability. The CMR scan took place within 2 months of the echocardiogram.

### Clinical features

The clinical characteristics of the individual patients are shown in Table S1 (see additional file 1). Of the 42 patients (age 48.7 ± 2.3 yrs, 60% male), 21 (50%) presented with dyspnoea, 8 (19%) with chest pain, 6 (14%) with palpitations, 2 (5%) with stroke, 1 (2%) with aortic regurgitation and 1 (2%) with recurrent pulmonary emboli. Two patients who presented with dyspnoea had a previous history of pulmonary embolism. One patient who presented with palpitations due to atrial fibrillation had suffered a left brachial artery embolism. Of the 6 patients who were asymptomatic, 1 patient had a family history of sudden cardiac death, 1 had a family history of sudden cardiac death and dilated cardiomyopathy, 1 had a family history of hypertrophic cardiomyopathy, 1 was referred following the finding of left ventricular hypertrophy (LVH) on echocardiography and 2 were suspected as having NC on echocardiography, having presented following a screening medical examination.

Coronary angiography on the 8 patients who presented with chest pain revealed non-obstructive ( < 50% stenoses in the three main epicardial coronary arteries) coronary heart disease in 1 patient and normal, angiographically smooth coronary arteries in 7 patients.

### ECG and echocardiography

The 12-lead ECG was abnormal in 34 (81%) patients. A LBBB was present in 14 (33%), a RBBB in 2 (5%) and a delta wave (Wolf-Parkinson-White syndrome) in 1 (2%) patient. Voltage features of LVH were observed in 16 (38%) and a non-specific intraventricular conduction defect in one patient. Isolated lateral T wave inversion (leads V_4_–V_6_, I, and aVL) was seen in one patient. Atrial fibrillation was observed in 12/42 (29%), which was intermittent in 8 and permanent in 4. One patient with Wolf-Parkinson-White syndrome presented with a supraventricular tachycardia.

At the point of referral for CMR, transthoracic echocardiograms were abnormal in all patients. Features of LVH were observed in 29/42 (69%) and an LVEF ≤ 40% in 9/42 (21%). The suspicion of NC was raised in 4/42 (10%) echocardiograms.

### Cardiovascular magnetic resonance

Patients who presented with dyspnoea had higher left ventricular end-diastolic (LVEDV) and systolic (LVESV) volumes (both p < 0.0001) and a lower left ventricular ejection fraction (LVEF, p < 0.0001) than age-matched healthy controls (see table S2 in additional file 2). Out of the 42 patients with NC, 16 (38%) had a LVEF < 40%. Patients who did not present with dyspnoea also had higher LVEDV than healthy controls (p = 0.0469), but differences in LVESV and LVEF were not statistically significant.

**Figure 3:**
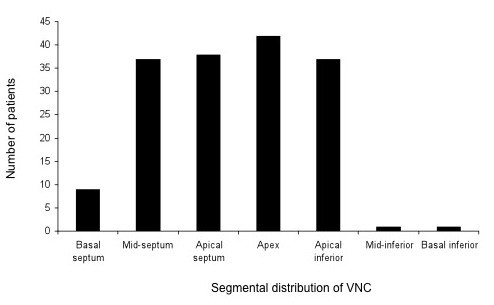
**Distribution of non-compacted myocardium according to myocardial segments**.

**Figure 4:**
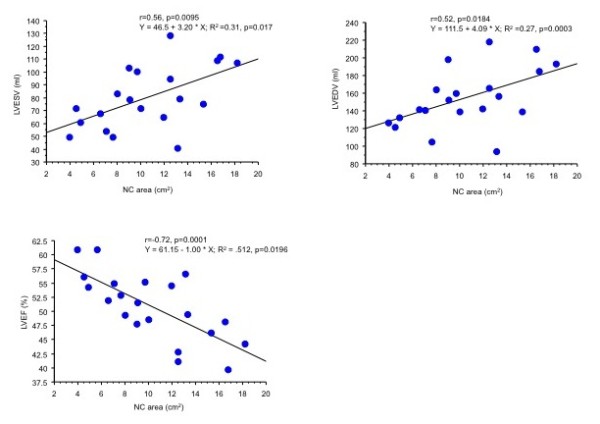
**Scattergrams of non-compacted (NC) area against left ventricular end-diastolic (LVEDV) and end-systolic (LVESV) volumes, and left ventricular ejection fraction (LVEF) in patients with left ventricular ventricular non-compaction who did not present with dyspnoea**.

As shown in Figure 3, NC myocardium was most frequently seen at the apex, followed by the apical septum, mid-septum, apical inferior wall, basal septum, mid-inferior and basal inferior walls. No significant differences emerged in the x:y ratio or NC area between patients presenting with dyspnoea and those without (see table S2 in additional file 2). In analyses of the whole study group, no correlation emerged between NC area and either LVEDV or LVESV. In the 21 patients without dyspnoea, however, NC area correlated positively with LVEDV (r = 0.52, p = 0.0184) and LVESV (r = 0.56, p = 0.0095) and negatively with LVEF (r = -0.72, p = 0.0001) (Figure 4).

## Discussion

We have shown that NC is associated with asymptomatic LVSD, heart failure dysrrhythmias, thromboembolic events and chest pain. Most patients in this study had abnormal ECGs and all had abnormal echocardiograms. Even patients who did not present with dyspnoea had higher LVEDVs than controls. It could be argued that these associations of NC are coincidental, as trabeculations are common even in normal hearts. [19] The fact that these patients presented with either symptoms or significant abnormalities on ECG, echocardiography and/or CMR, however, does suggest that NC contributes to pathogenesis. Anatomical correlation of suspected cases of NC could improve the diagnostic utility of investigations.

There is little consensus on the diagnostic criteria of NC. With respect to clinical criteria, Petersen et al have stipulated that the typical appearances of NC on CMR have to be accompanied by NC in a first degree relative, a neuromuscular disorder or complications, such as systemic embolisation or regional wall motion abnormalities. [11] Implicit in this approach, however, is the definition of NC *per se *as a distinct familial cardiomyopathy, rather than a pathological phenomenon that can occur in the absence of a family history. In this respect, a family history of cardiomyopathy is not always present in adults [7,9] or children [8] with NC. In the present study, no patient was found to have a family history of NC and only 11% had a family history of other cardiac conditions, such as dilated cardiomyopathy, sudden cardiac death or hypertrophic cardiomyopathy. Whilst our findings suggest that NC *per se *is not necessarily familial, they raise the possibility of a genetic link between NC, dilated cardiomyopathy, sudden cardiac death and hypertrophic cardiomyopathy. Other studies have shown that familial NC encompasses abnormalities that overlap with those seen in families with dilated cardiomyopathy. [9] Whether or not these two conditions are genetically linked or whether they share a common etiology remains unclear.

The extent of NC has also been adopted as a diagnostic criterion for NC. Several authors have stipulated diagnostic thresholds for NC using various measures of NC [2,6,7,11] Using either the x:y ratio proposed by Chin et al [6] or the NC area on the two-chamber view, we have found no difference in the extent of NC between patients presenting with dyspnoea and those without. Interestingly, significant clinical problems occurred even in patients with mild degrees of NC. An example is patient #28, an 18 year old male with a history of multiple strokes, intermittent atrial fibrillation, an ejection fraction of 36%, a mild degree of NC and an otherwise structurally normal heart on both echocardiography and CMR.

In this study, 38% of patients with NC had a CMR-derived LVEF < 40%. These findings are consistent with other studies showing that adult NC is frequently associated with LVSD, with reports ranging from 63% to 82%. [3,6,7,9] In the whole study group, no correlation emerged between the x:y ratio or the NC area on the two-chamber view and LVEF. In patients who did not present with dyspnoea, however, there was an inverse correlation between NC area and LVEF, suggesting that disruption of myocardial architecture associated with NC [15] contributes to LVSD, at least up to the point that the patient develops dypsnoea.

Some authors have underlined the tendency to interpret the appearance of NC on transthoracic echocardiography as concentric LVH, [20] hypertrophic cardiomyopathy [21] or an apical tumor. [22] In this study, NC was suspected in 10% of transthoracic echocardiograms performed in referring centres. The majority had been reported as showing concentric LVH or LVSD. This supports the finding of other authors [11,20,22] that CMR is superior to transthoracic echocardiography in detecting NC. The relatively low sensitivity of transthoracic echocardiography for detecting LVNC was not explored in this study.

The high incidence of symptomatic cases presented in this series is consistent with work from other tertiary referral centres. [7,9,23] It is likely that the left ventricular impairment is responsible for a high proportion of the symptoms. The higher incidence of NC in males compared with females is difficult to interpret in this retrospective study, and is not statistically significant. It may reflect referral bias or possibly, a higher prevalence of NC in males.

All patients in our series had abnormalities of the resting ECG, including voltage signs of left ventricular hypertrophy, inverted T waves in the chest leads, ST segment depression, axis deviation and intraventricular conduction abnormalities. A left bundle branch block was observed in 33%. In one case, NC was associated with Wolf-Parkinson White syndrome, which has been reported in 15% of the childhood form of NC. [8]

Arrhythmias occur frequently is association with NC. We, as others, [3,7] have found an association between NC and atrial fibrillation, which was the presenting problem in 29% of patients. Although the largest study of NC in children showed no association with ventricular tachycardia, [8] other studies of adult NC found ventricular tachycardia in 20 to 40% patients. [9,6] No association with ventricular tachycardia was found in the present study, although screening with Holter monitoring was not undertaken.

Thromboembolic events have been reported in 21% to 38% of patients with NC. [3,6,7] In this study, some patients suffered a stroke, pulmonary embolism or a peripheral embolism. These events may be attributable to atrial fibrillation, which was found in 2/4 of these patients. Alternatively, thromboembolic events may be due to thrombus formation within the intertrabecular recesses in NC myocardium. [6]

Chest pain was the presenting symptoms in 19% of patients. Angiography revealed non-obstructive coronary heart disease in one patient and normal coronary arteries in 7 patients. In this respect, reduced coronary blood flow reserve [24] and subendocardial perfusion defects [25,26] are known to occur in association with NC. These are thought to relate to failure of the coronary circulation to grow with the myocardial mass and/or compression of the intramural capillaries by hypertrophied myocardium. [25] Severe hyperplasia of the vascular media [15] may also be relevant. The possible link between NC, ischemia and chest pain, however, remains unexplored.

One of the limitations of this study is that the data presented are derived from opportunistic investigations, prompted by the clinical presentation and the results of other investigations. It is possible, therefore, that the prevalence of dysrhythmias, for example, is higher than that observed in this study. We have not undertaken family screening and we cannot, therefore, discount the presence of clinically occult NC in patients' relatives. The echocardiograms were not systematically re-studied after the CMR scan; however, this study suggests that CMR allows greater detection of NC.

A further limitation of the study is that some of the patients included in this study had been referred specifically from other centres for CMR. The majority of patients were seen at this centre, where CMR is a routine investigation. This study must be regarded as including a very selected group of patients, and as such this study may not be generalisable to all patients with NC.

## Conclusion

This study shows that NC is associated with ECG abnormalities, dysrrhythmias, thromboembolic events, chest pain, asymptomatic LVSD and heart failure. Some patients have a family history of cardiomyopathy and/or sudden cardiac death. Its diagnosis is most adequately made using CMR, which allows identification as well as quantification of NC. Further studies are needed to determine whether the changes in myocardial architecture associated with non-familial NC is causally related to thrombogenesis, arrhythmogenesis and LVSD.

## Abbreviations

CMR: cardiovascular magnetic resonance; ECG: electrocardiogram; LVEDV: left ventricular end diastolic volume; LVEF: left ventricular ejection fraction; LVESV: left ventricular end systolic volume; LVH: left ventricular hypertrophy; LVSD: left ventricular systolic dysfunction; NC: non-compaction.

## Competing interests

The authors declare that they have no competing interests.

## Authors' contributions

ZRY wrote the manuscript and organised data analysis. PF re-drafted manuscript. SC, HM, KK, NUHM were involved in analysis of the data. FL co-wrote manuscript. All author read and approved the final version of the manuscript.

## Pre-publication history

The pre-publication history for this paper can be accessed here:



## Supplementary Material

Additional File 1 **Table S1**. Summary of clinical, electrocardiographic, echocardiographic and cardiovascular magnetic resonance characteristics of the study group. Click here for file

Additional File 2 **Table S2**. Cardiovascular magnetic resonance characteristics of the study and control groups. Click here for file
