# Supplementary material for: Left ventricular non-compaction: clinical features and cardiovascular magnetic resonance imaging
Source: BMC Cardiovasc Disord. 2009 Aug 9;9:37. doi: 10.1186/1471-2261-9-37 (PMC2743643; doi:10.1186/1471-2261-9-37)
Supplement: Additional File 1 — Table S1. Summary of clinical, electrocardiographic, echocardiographic and cardiovascular magnetic resonance characteristics of the study group. [file 1471-2261-9-37-S1.doc]

**Table 1.** Summary of clinical, electrocardiographic, echocardiographic and cardiovascular magnetic resonance characteristics of the study group.

| # | Sex | Age | **Clinical presentation** | Other conditions | FH | Rhythm | ECG | Echo | x:y  ratio | NC area | LVEF |
| --- | --- | --- | --- | --- | --- | --- | --- | --- | --- | --- | --- |
| 1 | M | 68 | Dyspnoea |  |  | SR | RBBB | LVH | 0.37 | 4.4 | 48 |
| 2 | M | 37 | Asymptomatic |  | SCD | SR | RBBB | LVH | 0.21 | 12.5 | 41.1 |
| 3 | F | 63 | Chest pain |  |  | PAF | Normal | LVH | 0.25 | 4.0 | 61 |
| 4 | F | 65 | Dyspnoea |  |  | AF | LBBB | LVSD | 0.24 | 11.7 | 25.3 |
| 5 | M | 30 | Asymptomatic |  | DCM and SCD | SR | Normal | LVH | 0.27 | 12.5 | 42.9 |
| 6 | M | 18 | Palpitations | WPW |  | SVT | WPW | LVH | 0.20 | 18.2 | 44.3 |
| 7 | M | 53 | Chest pain | CHD |  | SR | LVH | LVH | 0.27 | 15.3 | 46.2 |
| 8 | M | 62 | Dyspnoea |  |  | PAF | LBBB | LVSD | 0.24 | 13.5 | 45.2 |
| 9 | M | 40 | Chest pain |  |  | SR | LVH | LVH | 0.17 | 16.7 | 39.7 |
| 10 | M | 40 | Chest pain |  | DCM | SR | LVH | LVH | 0.22 | 13.3 | 49.5 |
| 11 | M | 67 | Dyspnoea | OSAS |  | SR | Normal | LVH | 0.21 | 12.8 | 51.9 |
| 12 | F | 41 | Chest pain, dyspnoea |  |  | SR | LBBB | LVH | 0.47 | 8.0 | 32.6 |
| 13 | F | 76 | Dyspnoea |  |  | SR | LBBB | LVSD | 0.25 | 9.9 | 25.5 |
| 14 | M | 42 | Dyspnoea | PE | DCM | SR | LBBB | LVSD | 0.30 | 5.6 | 14.7 |
| 15 | F | 57 | Chest pain |  |  | SR | LVH | LVH | 0.31 | 13.1 | 56.7 |
| 16 | M | 56 | Dyspnoea |  |  | PAF | LBBB | LVSD | 0.30 | 11.9 | 15.5 |
| 17 | F | 36 | Dyspnoea | PE |  | PAF | LVH | LVSD | 0.17 | 18.7 | 31.2 |
| 18 | M | 53 | Palpitations |  |  | PAF | LVH | LVH | 0.46 | 7.6 | 52.9 |
| 19 | M | 59 | Dyspnoea |  |  | AF | T wave inversion | LVH | 0.31 | 19.5 | 24.2 |
| 20 | F | 50 | Palpitations |  |  | PAF | LVH | LVH | 0.53 | 12.0 | 54.6 |
| 21 | M | 53 | Dyspnoea | Hypertension |  | SR | LBBB | LVSD | 0.36 | 10.6 | 42.1 |
| 22 | M | 47 | Dyspnoea |  |  | SR | LBBB | LVH | 0.23 | 11.6 | 31.2 |
| 23 | M | 52 | Dyspnoea |  |  | SR | LBBB | LVSD | 0.47 | 11.8 | 11.4 |
| 24 | M | 16 | Palpitations |  |  | SR | LVH | LVH | 0.17 | 8.0 | 49.3 |
| 25 | M | 40 | Recurrent PE |  |  | SR | LVH | LVH | 0.35 | 4.9 | 54.3 |
| 26 | F | 31 | Stroke |  |  | SR | LVH | LVH | 0.27 | 7.1 | 55 |
| 27 | F | 40 | Asymptomatic |  |  | SR | LVH | LVH | 0.38 | 6.6 | 51.9 |
| 28 | M | 18 | Dyspnoea | Multiple strokes |  | PAF | LVH | LVH | 0.29 | 9.1 | 36.4 |
| 29 | F | 71 | Dyspnoea |  |  | SR | LBBB | LVH | 0.53 | 7.8 | 24.4 |
| 30 | F | 19 | Asymptomatic |  | HCM | SR | Normal | NC? | 0.24 | 10.0 | 48.6 |
| 31 | F | 63 | Dyspnoea |  |  | SR | Normal | NC? | 0.18 | 4.6 | 66.3 |
| 32 | M | 58 | Asymptomatic | Hypertension |  | SR | LVH | LVH | 0.31 | 16.5 | 48.2 |
| 33 | F | 59 | Dyspnoea |  |  | SR | LVH | LVSD | 0.30 | 10.6 | 32.6 |
| 34 | F | 35 | Dyspnoea |  |  | SR | Normal | NC? | 0.26 | 9.7 | 35 |
| 35 | F | 54 | Chest pain |  |  | PAF | Normal | LVH | 0.30 | 5.6 | 61 |
| 36 | M | 49 | Aortic regurgitation | HCM |  | SR | LVH | LVH | 0.44 | 9.1 | 51.5 |
| 37 | M | 59 | Dyspnoea |  |  | SR | LBBB | NC? | 0.20 | 12.5 | 43.1 |
| 38 | M | 48 | Palpitations |  |  | AF | Normal | LVH | 0.30 | 9.0 | 47.8 |
| 39 | F | 57 | Dyspnoea |  |  | SR | LBBB | LVH | 0.35 | 6.4 | 11.3 |
| 40 | M | 76 | Dyspnoea |  |  | SR | LBBB | LVH | 0.21 | 15.9 | 8.4 |
| 41 | F | 56 | Palpitations | Brachial artery embolism |  | AF | LBBB | LVH | 0.29 | 4.5 | 56.1 |
| 42 | M | 32 | Chest pain |  |  | SR | LVH, NSIVCD | LVH | 0.13 | 9.7 | 55.2 |

FH=family history, Echo=echocardiographic findings, x:y ratio= ratio of distance between epicardial surface and trough of the recesses (x) to the distance between the epicardial surface and the peak of the trabeculations (y) (see text), NC area = planimetered of the area of non-compacted myocardium on the two-chamber view in cm^2^, SR = sinus rhythm, PAF = paroxsysmal atrial fibrillation, AF = permanent atrial fibrillation, SVT = supraventricular tachycardia, LVEF = left ventricular ejection fraction (%), RBBB = right bundle branch block, LBBB = left bundle branch block, LVSD = left ventricular systolic dysfunction defined as an LVEF≤40%, NC? = suspected left ventricular non-compaction, NSIVCD = non-specific intraventricular conduction defect, DCM = dilated cardiomyopathy, SCD = sudden cardiac death, WPW = Wolf-Parkinson-White syndrome, CHD = coronary heart disease, OSAS = obstructive sleep apnea syndrome, PE = pulmonary embolism, HCM = hypertrophic cardiomyopathy.
