# Supplementary material for: Left ventricular non-compaction: clinical features and cardiovascular magnetic resonance imaging
Source: BMC Cardiovasc Disord. 2009 Aug 9;9:37. doi: 10.1186/1471-2261-9-37 (PMC2743643; doi:10.1186/1471-2261-9-37)
Supplement: Additional File 2 — Table S2. Cardiovascular magnetic resonance characteristics of the study and control groups. [file 1471-2261-9-37-S2.doc]

**Table 2.** Cardiovascular magnetic resonance characteristics of the study and control groups.

**Healthy controls** **NC *p***

**Group A B C B vs A C vs A C vs B**

**No dyspnoea** **Dyspnoea**

*N*  22 21 21

Age, yrs 48.9 ± 3.5 41.6 ± 3.1 55.2 ± 3.2 NS 0.051 NS

Non-compaction indices

NC area, cm^2^ -- 10.3 ± 0.9 10.7 ± 0.9 -- -- NS

x:y ratio -- 0.29 ± 0.0 0.29 ± 0.0 -- -- NS

LV function

LVESV, cm^3^ 52.4 ± 3.6 80.1 ± 5.4 165.4 ± 23.6 NS <0.0001 <0.0001

LVEDV, cm^3^ 113.3 ± 6.0 154.5± 7.4 224.6 ± 22.9 0.0469 <0.0001 0.0012

LVEF, % 54.4 ± 1.3 50.3 ± 1.3 31.3 ± 3.2 NS <0.0001 <0.0001

NC=left ventricular non-compaction, NC area = area of non-compacted myocardium on the two-chamber long axis LV view, x:y ratio: the ratio of distance between epicardial surface and trough of recesses (x) to the distance between the epicardial surface and the peak of trabeculation (y) [6], LVESV = left ventricular end-diastolic volume, LVEDV = left ventricular end-diastolic volume, LVEF = left ventricular ejection fraction
